# Analysis on Damage and Mechanical Properties of Ballastless Track in a Tunnel after a Fire

**DOI:** 10.3390/ma15196712

**Published:** 2022-09-27

**Authors:** Hujun Ma, Wei Chen, Xiang Li, Qingyuan Xu, Ping Lou, Chencai Tong

**Affiliations:** 1 School of Civil Engineering, Central South University, Changsha 410075, China; 2 National Engineering Research Center of High-Speed Railway Construction Technology, Central South University, Changsha 410075, China; 3 School of Civil Engineering, Sun Yat-sen University, Zhuhai 519082, China; 4 T.Y.Lin International Engineering Consulting (China) Co., Ltd., Chongqing 401121, China

**Keywords:** uniaxial compressive strength, shear strength, high-temperature damage constitutive equation for concrete, temperature field, damage of ballastless track

## Abstract

In order to explore the damage and mechanical properties of ballastless track after a fire, the uniaxial compressive strength, shear strength, peak strain, and elastic modulus changes due to temperature were obtained through uniaxial compressive and shear tests of concrete after exposure to high temperatures. The test results showed that with increases in temperature, the uniaxial compressive strength, shear strength, and elastic modulus of concrete all presented a decreasing trend, while the peak strain had an increasing trend. Then, based on the classical damage theory model and the strength probability distribution function of concrete micro-units, the high-temperature damage constitutive equation for concrete was established, and the compressive stress–strain curve of concrete after exposure to high temperature was reproduced. Finally, using the CFD numerical simulation software, the temperature field of a ballastless track structure in a tunnel during a fire was obtained, and the temperatures at different positions of ballastless track bed were acquired. Combined with the high-temperature damage constitutive equation for concrete deduced from tests and theoretical analysis, the strength and damage values of the ballastless track bed at different positions after a tunnel fire were obtained.

## 1. Introduction

Concrete is one of the most important building materials, and its mechanical properties are an important factor in determining its service performance. After exposure to a high-temperature environment, the mechanical properties of concrete are very different from those at normal temperatures [1,2,3,4]. The mechanical properties of concrete after exposure to high temperatures have a great influence on the performance evaluation and reinforcement of structures after a fire [5,6,7,8]. Many scholars have studied the compressive strength, shear strength, elastic modulus, damage and so on of concrete after exposure to high temperatures. Toric et al. [9] carried out a test by heating high-strength concrete samples to a high target temperature; the test analyzed compressive strength, tensile strength, and the tangent and secant elastic modulus. Three groups of standard concrete test specimens with different water contents were tested for compressive strength by Xu et al. [10], and it was concluded that high water content could improve residual compressive strength when the temperature was lower than 200 °C. Knobloch et al. [11] investigated the uniaxial performance of concrete at elevated temperatures under cyclic compressive loading, and confirmed the suitability of the damage-plasticity modelling concept for concrete under uniaxial compressive stress at elevated temperatures. Aziz et al. [12] studied the high-temperature performance of a low-strength concrete specimen, and it was found that the ductility and residual Poisson’s ratio of the concrete increased with the increase in heating temperature after heating at 700 °C; the elastic modulus of the concrete decreased sharply after heating at 300 °C; and the concrete lost more than 80% of its stiffness after exposure to 600 °C. Le et al. [13] established a simple formula for the stress–strain-temperature relationship of concrete, which was consistent with the stress–strain curve of concrete in Eurocode 2 used for the heating phase. Nuaklong et al. [14] studied the confounding effect of multi-walled carbon nanotubes (MWCNTs) and polypropylene (PP) fibers on the mechanical and fire resistance of Portland cement mortar, and the strength of the mortar was measured at different temperatures below 1000 °C. Sukontasukkul et al. [15] studied various effects of high temperature on the mechanical properties of fiber-reinforced concrete (FRC), and conducted flexural toughness tests on two types of concrete (plain concrete and fiber-reinforced concrete) according to ASTM C1018. Sukontasukkul et al. [16] directly determined the damage to concrete under static and impact compressive loads using damage mechanics theory. Two methods based on the variation of (1) elastic modulus (E) and (2) strain rate (ε) were used.

In recent years, tunnel fire accidents have occurred frequently, often causing serious casualties and property losses. Tunnel fire research [17,18,19,20,21] has become an important issue in the field of fire research. Many scholars have simulated tunnel fires through different tests, models, and different software such as FDS and FLUENT under the influence of factors such as fire scale, fire environment, and tunnel types to obtain the structural temperature field, smoke flow rules and so on in the tunnel. Qu et al. [22] and Lu et al. [23] used the conservation law, the theories of high-temperature gas dynamics, and fluid mechanics to set up the physical and mathematical turbulence flow field model and simulated a tunnel fire and its smoke flow. Lu et al. [24] studied the temperature distribution in curved tunnels using a numerical simulation method, and the results showed that the temperature was slightly higher for a concave wall than a convex wall for the distant positions from the fire source, which was different from that in a straight tunnel. Li et al. [25] carried out 1:10 reduced-scale tests for branched tunnels to investigate the longitudinal fire location effects on thermal smoke temperature distribution beneath the ceiling. Qu et al. [26] established physical and mathematical turbulence flow field models to numerically simulate railway tunnel fire and smoke flow; the results showed that the temperature distribution of laminar flame, smoke concentration, and flow velocity can be represented by fully developed smoke flow in the downwind direction. Sukontasukkul et al. [27] studied the effect of fire on the flexural properties and residual strength of ordinary concrete and fiber-reinforced concrete and tested the bending load of the sintered sample to determine its toughness and residual strength.

These studies have not only greatly deepened the understanding of the damage and mechanical properties of concrete after exposure to high temperatures but have also achieved fruitful work on smoke flow and temperature fields of tunnel fires. However, there is still a lack of targeted research on the temperature field distribution of the ballastless track and the damage and mechanical properties of the concrete track bed in a tunnel after a fire. Therefore, this paper carried out tests of the mechanical properties of concrete after exposure to high temperatures. Starting from the classical damage theory model and the strength probability distribution function of concrete micro-units, the high-temperature damage constitutive equation for concrete was established. Then, the temperature field of the ballastless track structure in a tunnel during a fire was simulated using the computational fluid dynamics (CFD) numerical software FLUENT, and finally the damage and mechanical properties of a ballastless track structure in a tunnel after a fire were obtained.

## 2. Uniaxial and Shear Tests

### 2.1. Test Preparation

The materials and mix properties used in preparing the concrete related to the experimental part of this paper were as shown in Table 1 [28].

**Figure 1:**
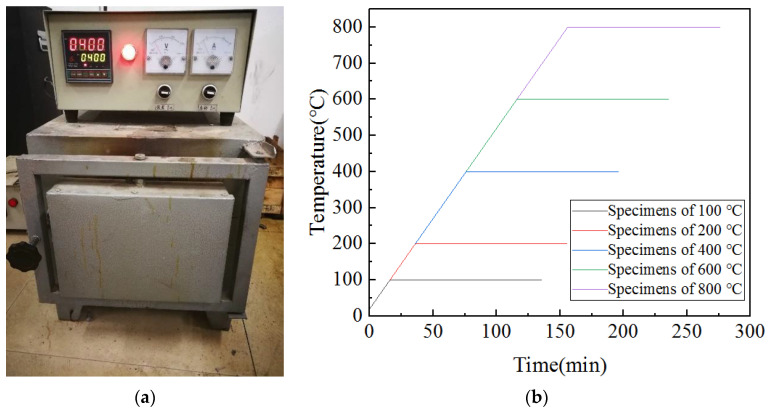
Heating process: (**a**) the resistive heating furnace (model: KSY-12-T); (**b**) heating process.

The concrete was made into a cube specimen of 150 mm × 150 mm × 150 mm and cured in a standard curing room for 28 days after demolding. Before the mechanical properties were tested, a resistive heating furnace (model: KSY-12-T), produced by Changsha Changcheng Electric Furnace Factory, was used to heat the concrete specimens [29], as shown in Figure 1. During heating, all specimens were heated to a predetermined high temperature (100 °C, 200 °C, 400 °C, 600 °C, or 800 °C) in the heating furnace at a rate of 5 °C/min, and then the temperature was maintained for 2 h to make sure the specimens were heated evenly. Next, the high-temperature specimens were cooled by air cooling, that is, the specimens were placed in a room-temperature environment until the temperature dropped to room temperature. During the heating process, the thermometer reading inside the heating furnace was read and recorded every 60 s, and the temperature change of the specimen during the heating process was obtained as shown in Figure 1b. Finally, the mechanical properties such as uniaxial compressive strength and shear strength were tested.

### 2.2. Test Method

**Figure 2:**
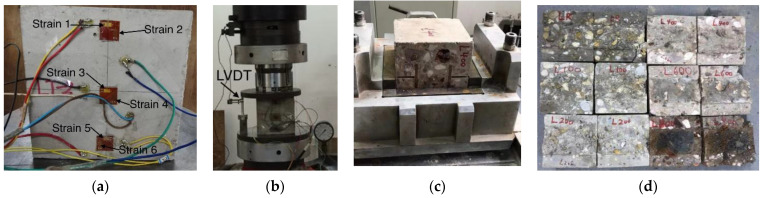
Photos of test detail: (**a**) location of strain gauges; (**b**) location of LVDT; (**c**) shear test; (**d**) failure specimens.

Compressive strength and shear strength are important indicators to measure the mechanical properties of concrete, which are greatly affected by temperature. In order to study the influence on the mechanical properties of concrete after exposure to high temperatures, uniaxial compressive and shear tests were carried out on the heated specimens at 20 °C, 100 °C, 200 °C, 400 °C, 600 °C, and 800 °C. The compression test used a uniaxial compression form; the location of strain gauges is shown in Figure 2a, and the location of the LVDT is shown in Figure 2b. The shear test adopted a direct shear form, as shown in Figure 2c, and the instrument was equipped with an LVDT. The shear test measured the peak shear force of a concrete specimen, which was then divided by the shear area to obtain the shear strength. In addition, in the uniaxial compression test and shear test, the force control mode was used, and the loading rate was 0.5 kN/s. On the tested concrete specimens, L represents low-strength concrete, and the number represents the temperature that concrete was subjected to during the test. For example, L600 expresses the low-strength concrete that was heated to 600 °C. In the tests for this paper, there were two types of concrete specimens for each temperature (20 °C, 100 °C, 200 °C, 400 °C, 600 °C, and 800 °C), one of which was used for the uniaxial compression test and one for the shear test, as shown in Figure 2d.

## 3. Test Results and Analysis

### 3.1. Stress–Strain Curve of the Uniaxial Compressive Test

**Figure 3:**
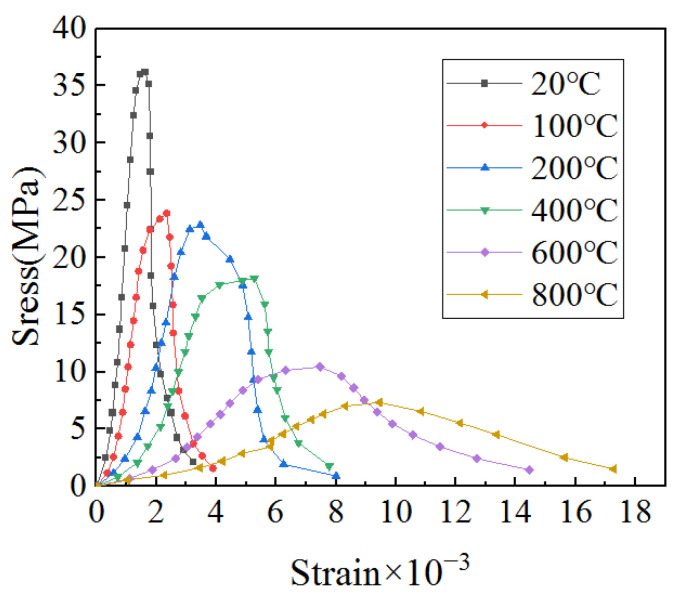
Stress−strain curve of concrete during the uniaxial compressive test after different heated temperatures.

In order to obtain a more accurate stress–strain curve, during the experiment the instrument could automatically record the force and displacement, and then divide the force by the loading area to obtain the stress and divide the displacement by the height to obtain the strain. The converted curve is the stress–strain curve, and the stress–strain curves of concrete specimens after different heated temperatures are shown in Figure 3. In addition, the peak stress and peak strain of specimens are shown in Table 2. It can be seen from Figure 3 and Table 2 that with the increase in temperature, the shape of the curve transitions from sharp to flat, indicating that the stress of the concrete gradually decreases while the strain gradually increases, resulting in damage inside the specimen, and high temperature will cause great damage to concrete.

### 3.2. Elastic Modulus

**Figure 4:**
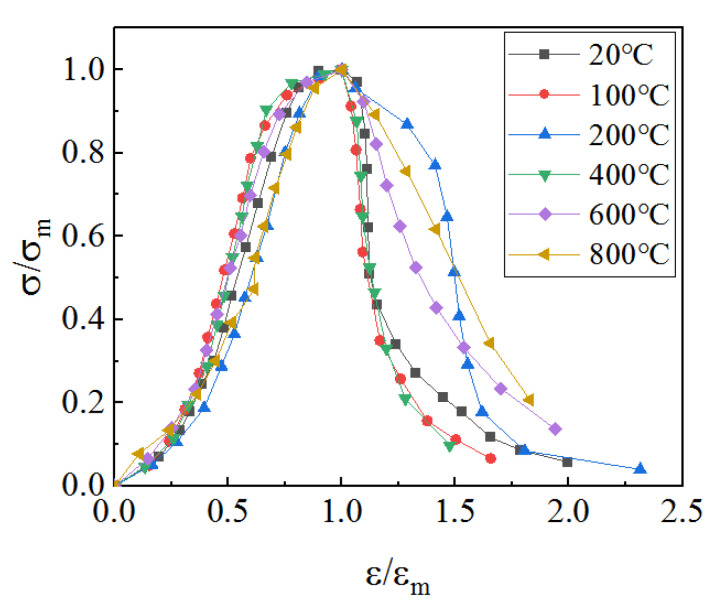
Full stress–strain curve.

In this paper, the stress–strain curves after different heated temperatures were normalized without dimensionality [30], that is, the strain on the abscissa was divided by the peak strain, the stress on the ordinate was divided by the peak stress, and then the full stress–strain curve was drawn as shown in Figure 4. It can be seen from Figure 4 that the full stress–strain curves after different heated temperatures basically overlap in the ascending segment before ε/ε_m_ equals 1, while the descending segment after ε/ε_m_ equals 1 has greater discreteness and has varying degrees of smoothness.

**Figure 5:**
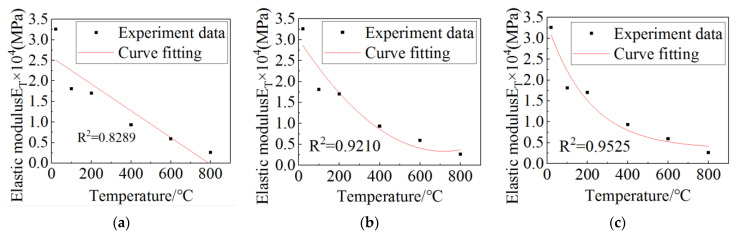
Fitting curve of temperature and elastic modulus: (**a**) linear fitting; (**b**) polynomial fitting; (**c**) exponential fitting.

At the same time, in order to quantitatively describe the relationship between elastic modulus and temperature, on the basis of obtaining the full stress–strain curve, stress and the corresponding strain at the ordinate of 0.4 on the full stress–strain curve were taken. Finally, the elastic modulus of the concrete specimens after different heated temperatures were obtained by dividing the strain by the stress obtained through the above method [31], as shown in Table 3. Linear, polynomial, and exponential fitting methods were used to fit the relationship between the elastic modulus and the temperature, and the results are shown in Figure 5. It can be seen that the elastic modulus of the concrete decreases exponentially after high-temperature heating and cooling. During fitting, the larger the R-square was, the higher the fitting accuracy was. By comparing and analyzing the R-square of various fitting methods, it can be concluded that the exponential fitting result is more accurate, and the fitting formula is shown as follows:

(1) $$E_{T}=3.0066\times e^{\frac{-T}{209.2671}}+0.35259$$

There are two limitations on the use of this formula: first, the type of concrete was ordinary concrete, and the concrete strength grade was C35; second, the test type was a uniaxial compression test.

### 3.3. Relationship between Uniaxial Compressive Strength and Temperature

**Figure 6:**
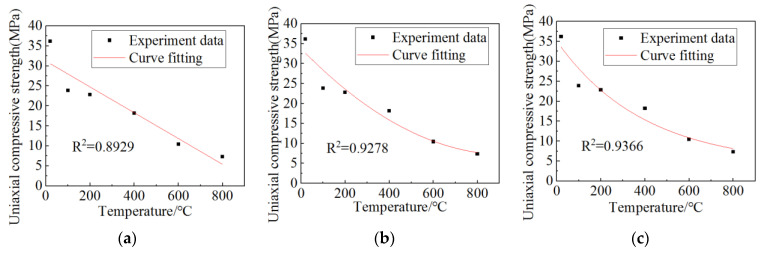
Fitting curve of the relationship between uniaxial compressive strength and temperature: (**a**) linear fitting; (**b**) polynomial fitting; (**c**) exponential fitting.

Table 4 lists the uniaxial compressive strength and the reduction rate of uniaxial compressive strength relative to 20 °C at different heated temperatures. It can be seen that with the increases in the temperature, the uniaxial compressive strength becomes lower and lower, while the compressive strength reduction rate becomes higher and higher. In order to characterize the correlation between uniaxial compressive strength and temperature, linear, polynomial, and exponential fitting methods were used to fit it, and the results are shown in Figure 6. When exponential fitting was adopted, the R-square was the largest and the fitting result was more accurate. The fitting formula is shown as follows:

(2) $$f_{c}\left(T\right)=31.08989\times e^{\frac{-T}{396.69649}}+3.99768$$

There are two limitations on the use of this formula: first, the type of concrete was ordinary concrete, and the concrete strength grade was C35; second, the test type was a uniaxial compression test.

### 3.4. Damage Value of Concrete after High-Temperature Heating

This paper uses two ways to evaluate the damage of concrete:

(1) Damage calculation using “residual strength” thermal damage model

Luo et al. [32] studied the compressive strength of concrete after exposure to high temperatures and proposed the “residual strength” thermal damage model, as shown in Formula (3). In combination with Formula (2), the “residual strength” thermal damage Formula (4) can be acquired.

(3) $$D_{1}\left(T\right)=1-\frac{f_{c}\left(T\right)}{f_{c0}}$$

(4) $$D_{1}\left(T\right)=1-\frac{31.08989\times e^{\frac{-T}{396.69649}}+3.99768}{36.15}$$

where *f_c_*_0_ is the uniaxial compressive strength of concrete at room temperature, f_c0_ = 36.15 MPa, and *D*_1_(*T*) is the damage value. The damage value *D*_1_(*T*) calculated by Formula (4) is shown in Table 4.

(2) Damage calculation using elastic modulus

Jiang et al. [33] determined the damage value *D*_2_(*T*) of concrete structure based on elastic modulus, as shown in Formula (5). In combination with Formula (1), the damage Formula (6) can be obtained.

(5) $$D_{2}\left(T\right)=1-\frac{E_{T}}{E_{0}}$$

(6) $$D_{2}\left(T\right)=1-\frac{3.0066\times e^{\frac{-T}{209.2671}}+0.35259}{3.23}$$

where *E_T_* is the elastic modulus of concrete after high-temperature heating, and *E*_0_ is the initial modulus of concrete at room temperature, *E*_0_ = 3.23 × 10^4^ MPa.

Similarly, the damage value calculated by Formula (6) is shown in Table 5. The results show that the damage to concrete increases nonlinearly with the increases in heating temperature.

### 3.5. High-Temperature Damage Constitutive Equation for Concrete

The damage to concrete is caused by the initiation, expansion, and connection of its internal cracks; both temperature loads and external forces may cause the generation of internal cracks, which constitute damage. Based on the classical damage theory model and the strength probability distribution function of concrete micro-units, an equation describing the whole process of concrete compressive damage at high temperature is obtained in this paper.

Taking the representative concrete volume unit used in the research and based on Lemaitre’s hypothesis of “equivalent strain”, the Formula (7) based on concrete damage mechanics can be obtained [34]:

(7) $$\sigma {=E}_{0}\left(1-D\right)\varepsilon$$

where σ is the nominal stress, ε is the nominal strain, *D* is the damage value, and $E_{0}=3.23\times 10^{4}$ MPa.

After the concrete was heated to high temperature, its mechanical properties weakened, and the stress–strain curves transitioned from sharp to flat, indicating that peak stress, uniaxial compressive strength, and elastic modulus decreased while peak strain increased. For this reason, the temperature-softening factor *K_T_* is introduced as shown in Formula (8):

(8) $$K_{T}=\frac{f_{c}\left(T\right)}{f_{c0}}$$

where *K_T_* is the temperature softening factor; *f_c_*(*T*) is the uniaxial compressive strength of concrete at high temperature, as shown in Formula (2); *f_c_*_0_ is the uniaxial compressive strength of concrete at room temperature, and *f_c_*_0_ = 36.15 MPa.

Therefore, when the influence of temperature is considered, the high-temperature damage constitutive equation for concrete can be described as follows [34]:

(9) $$\sigma {=E}_{0}\left(1-D\right)\varepsilon K_{T}$$

In the loading process of the uniaxial compressive test, it was assumed that the strength of each micro-unit in concrete followed the probability distribution of φ(ε), and the relationship between φ(ε) and damage value D is shown in Formula (10) [34]:

(10) $$\varphi \left(\varepsilon \right)=\frac{dD}{d\varepsilon }$$

The damage process of concrete can be divided into yield damage and fracture damage. When describing the damage evolution of concrete, the probability density of damage and fracture obey a three-parameter Weibull distribution, and the probability density of yield damage and fracture damage obedience is consistent. The formula is listed as follows [34]:

(11) $$\varphi \left(\varepsilon \right)=\frac{\beta }{\alpha }{\left(\varepsilon -\gamma \right)}^{\beta -1}\exp\left[-{\left(\frac{\varepsilon -\gamma }{\alpha }\right)}^{\beta }\right]$$

where α is the scale parameter, β is the shape parameter, and γ is the position parameter.

Combining Formulas (10) and (11), the expression of damage value D is shown in the following [34]:

(12) $$\begin{array}{r} D=\int_{0}^{\varepsilon }\varphi \left(x\right)dx=\frac{\beta }{\alpha }\int_{0}^{\varepsilon }{\left(x-\gamma \right)}^{\beta -1}\exp\left[-{\left[\frac{x-\gamma }{\alpha }\right]}^{\beta }\right]dx=-\exp\left[-{\left(\frac{x-\gamma }{\alpha }\right)}^{\beta }\right]|{}_{0}^{\varepsilon }\\ =1-\exp\left[-{\left(\frac{\varepsilon -\gamma }{\alpha }\right)}^{\beta }\right] \end{array}$$

(13) $$1-D=\exp\left[-{\left(\frac{\varepsilon -\gamma }{\alpha }\right)}^{\beta }\right]$$

In addition, in order to improve the accuracy of fitting, the corresponding coefficient *A* is added before the right side of the equal sign of Formula (13), so Formula (14) can be obtained by combining Formulas (2), (8), (9), and (13):

(14) $$\sigma {=E}_{0}\times \varepsilon \times A\times \exp\left[-{\left(\frac{x-\gamma }{\alpha }\right)}^{\beta }\right]\times \frac{31.08989\times e^{\frac{-T}{396.69649}}+3.99768}{36.15}$$

Through the regression and analysis of the test data, the values of A, γ, α, and β are listed in Table 6.

It can be seen from Table 6 that the value of A gradually decreases with the increase in temperature, while the values of γ and α gradually increase with the increase in temperature. In order to get the high-temperature damage constitutive equation for concrete at different heated temperatures besides 20 °C, 100 °C, 200 °C, 400 °C, 600 °C, and 800 °C, Formula (15) is obtained after fitting the relationship of values A, γ, α and temperature.

(15) $$\left\{ \begin{array}{l} A=0.00828\times T^{-0.02738}-0.00676\\ \gamma =0.00142\times T^{1.26}+1.318\\ \alpha =0.00014\times T^{1.568}+0.8194 \end{array}\right.$$

**Figure 7:**
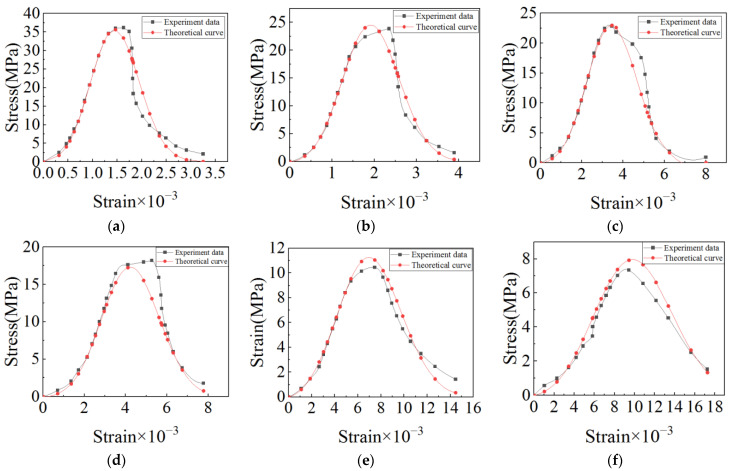
Comparison of test data and theoretical data: (**a**) 20 °C; (**b**) 100 °C; (**c**) 200 °C; (**d**) 400 °C; (**e**) 600 °C; (**f**) 800 °C.

Figure 7 shows the test results and the theoretical results from the high-temperature damage constitutive equation for concrete. It can be seen that the test data are close to the theoretical data. In the process of fitting by MATLAB, when the temperatures are 20 °C, 100 °C, 200 °C, 400 °C, 600 °C, and 800 °C, its R-square reaches 0.9851, 0.9930, 0.9914, 0.9945, 0.9955, and 0.9862 respectively. To sum up, the theoretical data calculated from the high-temperature damage constitutive equation is very close to the test data, which means the high-temperature damage constitutive equation can be used for reference in subsequent analysis.

### 3.6. Shear Strength

Shear strength is the basic mechanical property of concrete. In this paper, a shear test was carried out on concrete after being heated to a high temperature, and the test results are shown in Table 7.

**Figure 8:**
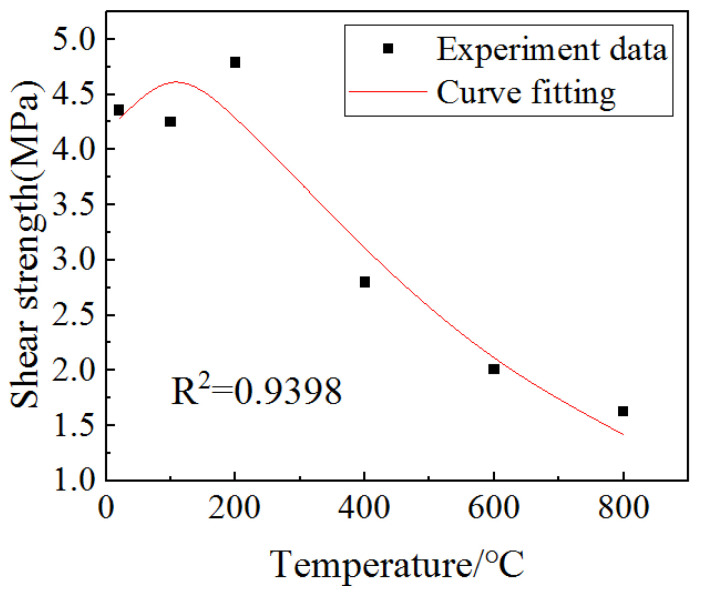
Fitting curve of the relationship between shear strength and heated temperature.

In addition, in order to express the relationship between shear strength and the temperature of the concrete, MATLAB was used to fit the data. The fitting Formula (16) was obtained, and the fitting results are shown in Figure 8.

(16) $$\tau \left(T\right)=7.184e^{-0.00203T}-3.153e^{-0.00917T}$$

It can be seen from Figure 8 that the shear strength of concrete decreases with the increase in temperature, mainly because the hydration of cement in concrete has been basically completed during the 28-day curing period. With the increase in temperature, concrete gradually dehydrates. Due to the different thermal expansion performances of the concrete’s internal composition, thermal stress is generated inside the concrete and can lead to cracks, so the shear strength of concrete decreases gradually.

## 4. Temperature Field and Mechanical Properties of a Ballastless Track Structure in a Tunnel during a Fire

During a fire, different positions of a ballastless track structure in a tunnel will have different temperatures. In order to obtain the temperature field, the CFD numerical simulation software FLUENT was used.

### 4.1. The Geometric Dimensions of the Model

**Figure 9:**
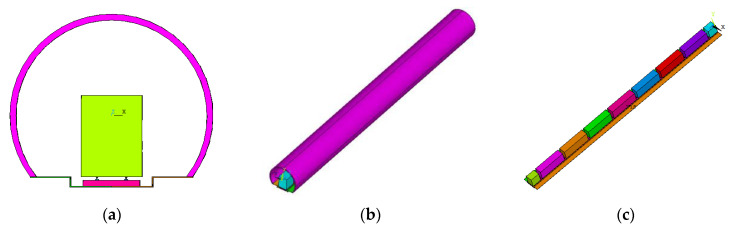
Screenshot of the model: (**a**) cross section of the model; (**b**) overall view of the model; (**c**) carriage layout of the model.

A numerical model was built. The total length of the tunnel was 100 m, the thickness and the inner diameter of the tunnel lining were 0.3 m and 4.9 m, respectively, and there were 7 carriages, each of which was 12 × 3 × 4 m^3^ in size, and the carriages were 1 m apart [35]. The substructure of this model consists of steel rail and a cast-in-place C35 concrete track bed. The cross-section width of the track bed was 2.8 m and the thickness was 0.3 m [36]. Figure 9 shows screenshots of the model, including the cross section, overall view, and carriage layout. In the model, the X, Y, and Z axes represents the width, thickness, and longitudinal direction of the bed, respectively.

### 4.2. Initial and Boundary Conditions of the Model

In this model, the grid size was 0.1 m × 0.1 m × 0.1 m within 20 m before and after the fire source, and the grid size was 0.4 m × 0.1 m × 0.1 m at other locations. It was assumed that in the initial state before the fire, the temperature of each element inside and outside the model was the same and equal to the temperature of the external environment, and the initial air pressure was a standard atmospheric pressure. In the model, the inlet boundary condition was the velocity inlet boundary, the outlet boundary condition was the pressure outlet boundary, the wind speed was set as 2 m/s, the heated boundary had heat convection and heat radiation, and the non-heated boundary only had heat radiation. In addition, the density of the concrete material was 2400 kg/m^3^, the specific heat capacity was 1100 J/(kg·K), and the thermal conductivity was 1.2 W/(m·k) [37], and this paper only carried out a simulation calculation for a designed fire of 15 MW [38]. It was assumed that the air inlet and outlet were respectively in the left and the right end of the tunnel, and the fire location was assumed to be in the third carriage from left to right.

### 4.3. Temperature Field of a Ballastless Track Structure in a Tunnel during a Fire

**Figure 10:**
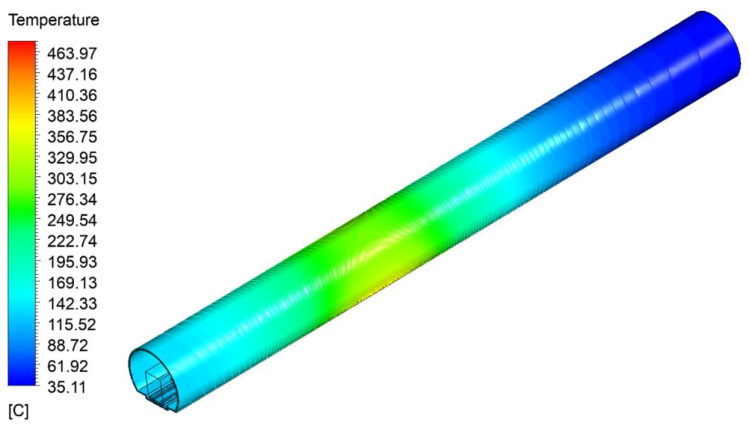
Temperature cloud of the tunnel vault along the longitudinal direction.

**Figure 11:**
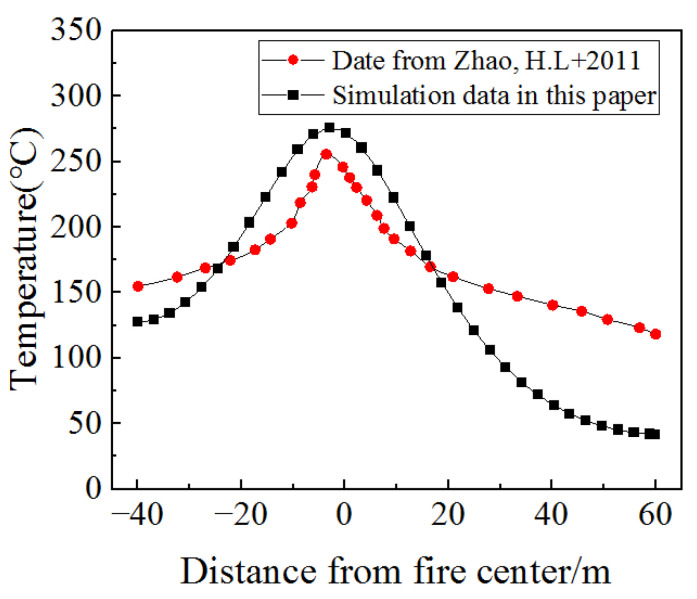
Comparison of the temperature range in the tunnel vault along the longitudinal direction between the reference [38] and the model in this paper.

The temperature cloud of the tunnel vault along the longitudinal direction is shown in Figure 10, and the comparison of the temperature range of the tunnel vault along the longitudinal direction between the reference [38] and the model in this paper is shown in Figure 11. It can be seen from Figure 11 that the temperature distribution of the tunnel vault along the longitudinal direction in the model was similar to the result from the reference [38]. Moreover, the temperature was the highest in the burning compartment, and the temperature near the two ends of the tunnel hole was lower. As for the specific value of the tunnel vault temperature, the maximum temperature value simulated in this paper and in the reference [38] was 275.54 °C and 255.58 °C, respectively, both of which are similar, so the correctness of the model in this paper is verified.

**Figure 12:**
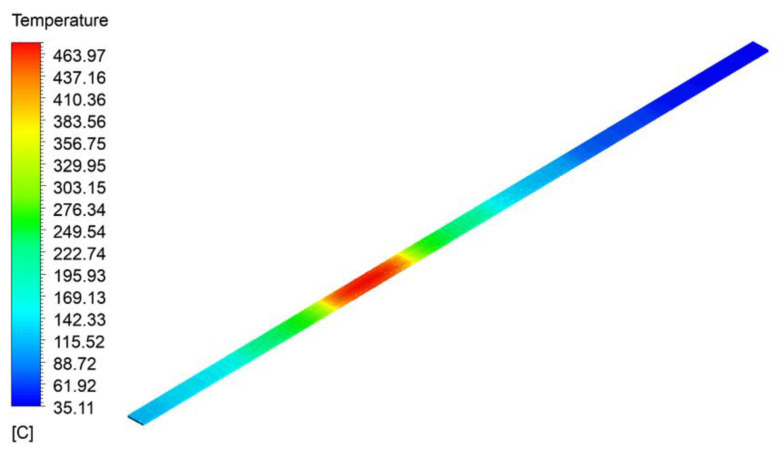
Overall temperature of the concrete track bed.

The overall temperature of the concrete track bed is shown in Figure 12. As can be seen from Figure 12, the temperature was the highest in the burning compartment, which was about 463.97 °C, and the lower temperatures were near the two ends of the tunnel hole; the temperature stratification is obvious in different locations.

**Figure 13:**
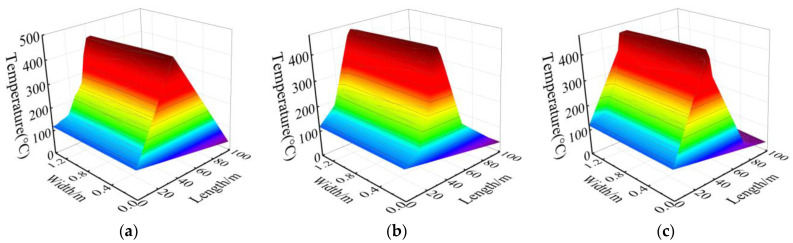
Temperature at different positions in the track bed: (**a**) surface; (**b**) middle; (**c**) bottom.

In addition, the overall temperature field of the cast-in-place C35 concrete track bed was symmetrical with respect to the X axis (width) of the track bed, so half of it was taken for study in the following. The temperature distribution at different positions in the track bed are shown in Figure 13. There is little difference in the temperature along the depth of the cast-in-place C35 concrete track bed. The highest temperatures at the positions of surface, middle, and bottom were 475.73 °C, 474.79 °C, and 472.63 °C, respectively, and the lowest temperatures were 39.24 °C, 39.23 °C, and 39.22 °C, respectively. However, the temperature variations were obvious along the width and longitudinal direction.

### 4.4. Mechanical Properties of a Ballastless Track Structure at Different Positions after a Tunnel Fire

**Figure 14:**
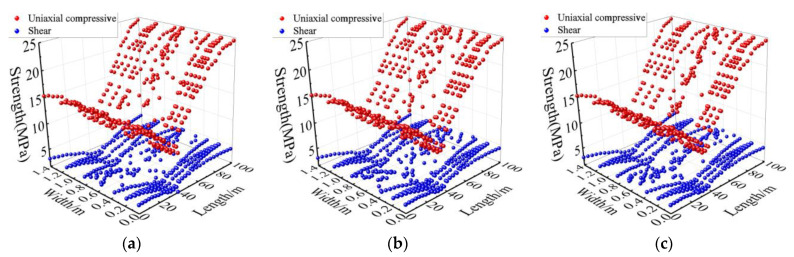
Uniaxial compressive strength and shear strength at different positions in the track bed: (**a**) surface; (**b**) middle; (**c**) bottom.

**Figure 15:**
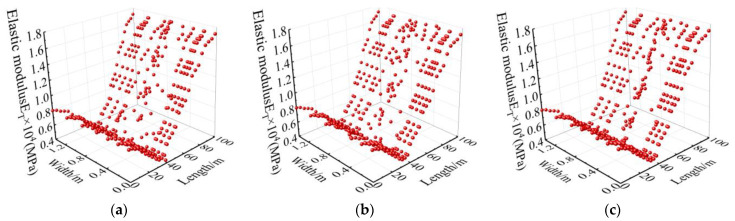
Elastic modulus at different positions in the track bed: (**a**) surface; (**b**) middle; (**c**) bottom.

**Figure 16:**
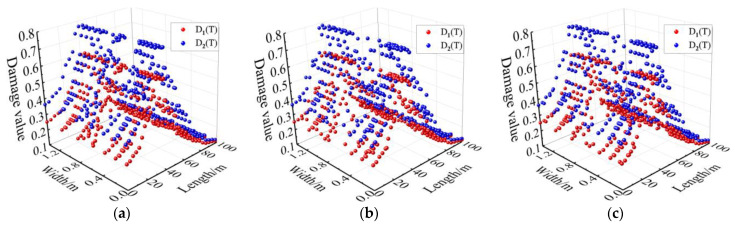
Damage value at different positions in the track bed: (**a**) surface; (**b**) middle; (**c**) bottom.

Combined with Figure 13 and the lab test results, the mechanical properties at different positions of the track bed are plotted in Figure 14, Figure 15 and Figure 16.

It can be seen from Figure 14, Figure 15 and Figure 16 that the mechanical properties of cast-in-place C35 concrete track bed are also different at different positions due to temperature variations. The compressive strength, shear strength, and elastic modulus of the track bed along the longitudinal direction show a V-shaped distribution, while the damage value shows an inverted V-shaped distribution. At the surface, middle, and bottom of the track bed, the smallest compressive strength was 13.37 MPa, 13.39 MPa, and 13.44 MPa, respectively; the smallest shear strength was 2.69 MPa, 2.70 MPa, and 2.71 MPa, respectively; and the smallest elastic modulus was 0.66 MPa, 0.66 MPa, and 0.67 MPa, respectively. The biggest damage value *D_1_*(*T*) and *D_2_*(*T*) at the surface, middle, and bottom of the track bed were 0.63 and 0.79, respectively. The higher the temperature, the lower the uniaxial compressive strength, shear strength, and elastic modulus, while the greater the damage value. Therefore, according to the high-temperature damage constitutive equation for concrete and the temperature field of the track bed, the strength and damage of the track bed at different positions after a tunnel fire can be accurately obtained, which can provide a theoretical basis for post-disaster assessment and reinforcement of the track structure.

## 5. Conclusions

▪ Taking 20 °C as the benchmark, when the temperature reaches 100 °C, 200 °C, 400 °C, 600 °C, or 800 °C, the uniaxial compressive strength, elastic modulus, peak strain, and shear strength of concrete changes after the increase in temperature as follows: the uniaxial compressive strength decreases by 34.00%, 36.85%, 49.68%, 71.09%, or 79.70%, respectively; the elastic modulus decreases by 43.03%, 46.44%, 71.21%, 81.42%, or 91.95%, respectively; the peak strain increases by 44.17%, 112.27%, 223.31%, 357.67%, or 479.75%, respectively; and the shear strength decreases by 2.52%, increases by 9.86%, or decreases by 35.78%, 53.90%, or 62.61%, respectively.
▪ Based on the mechanical testing of concrete after exposure to high temperatures, the high-temperature damage constitutive equation for concrete was established, and the stress–strain curves of concrete under uniaxial compressive after exposure to high temperatures were accurately simulated.
▪ FLUENT was used to simulate the temperature field of the cast-in-place C35 concrete track bed when a fire occurs in a tunnel. The surface of the track bed near the fire carriage had the highest temperature. Combined with the high-temperature damage constitutive equation for concrete and the formula for damage fitting, the strength and damage values of the track bed slab at different positions after a tunnel fire can be obtained quickly and accurately, which can provide a theoretical basis for post-disaster assessment and reinforcement of the track structure.

## Figures and Tables

**Table 1:** Mix proportions of concrete.

Mixtures	Dosage
Cement (kg/m^3^)	227
Water (kg/m^3^)	149
Fine sand (kg/m^3^)	795
Coarse aggregate (kg/m^3^)	1098
Limestone powder (kg/m^3^)	68
Slag (kg/m^3^)	44
Admixture (kg/m^3^)	6.4

**Table 2:** Peak stress and peak strain of the uniaxial compressive test after different heated temperatures.

Temperature/°C	20	100	200	400	600	800
Peak stress/MPa	36.15	23.86	22.83	18.19	10.45	7.34
Peak strain /10^−3^	1.63	2.35	3.46	5.27	7.46	9.45

**Table 3:** Elastic modulus of concrete after different heated temperatures.

Temperature/°C	20	100	200	400	600	800
Elastic modulus *E_T_* × 10^4^/MPa	3.23	1.84	1.73	0.93	0.60	0.26

**Table 4:** Uniaxial compressive strength and the reduction rate of uniaxial compressive strength.

Temperature/°C	20	100	200	400	600	800
Uniaxial compressive strength *f_c_*(*T*)/MPa	36.15	23.86	22.83	18.19	10.45	7.34
The reduction rate of uniaxial compressive strength/%	0.00	34.00	36.85	49.68	71.09	79.70

**Table 5:** Damage values *D*_1_(*T*) and *D*_2_(*T*).

Temperature/°C	20	100	200	400	600	800
Damage value *D*_1_(*T*)	0.0717	0.2210	0.3700	0.5757	0.6999	0.7749
Damage value *D*_2_(*T*)	0.0448	0.3136	0.5329	0.7532	0.8379	0.8705

**Table 6:** Parameters for high-temperature damage constitutive equation for concrete.

Temperature/°C	A	γ	α	β
20	0.0008687	1.258	0.745	2
100	0.0005413	1.641	1.015	2
200	0.0003475	2.973	1.810	2
400	0.0003178	3.701	2.092	2
600	0.0001827	5.668	4.242	2
800	0.0001223	8.011	5.920	2

**Table 7:** Shear strength of concrete after different heated temperatures.

Temperature/°C	20	100	200	400	600	800
Shear strength/MPa	4.36	4.25	4.79	2.80	2.01	1.63

## Data Availability

Not applicable.
